# Accelerating Disease Model Parameter Extraction: An LLM-Based Ranking Approach to Select Initial Studies For Literature Review Automation

**DOI:** 10.3390/make7020028

**Published:** 2025-03-26

**Authors:** Masood Sujau, Masako Wada, Emilie Vallée, Natalie Hillis, Teo Sušnjak

**Affiliations:** 1 School of Veterinary Science, Massey University, Palmerston North 4442, New Zealand; 2 School of Mathematical and Computational Sciences, Massey University, Auckland 0632, New Zealand

**Keywords:** large language models in systematic reviews, automated AI literature screening, zero-shot relevancy ranking, climate-sensitive zoonotic disease modelling, information retrieval in medical literature, systematic literature review automation, biomedical text mining for disease tracking, AI-assisted disease surveillance

## Abstract

As climate change transforms our environment and human intrusion into natural ecosystems escalates, there is a growing demand for disease spread models to forecast and plan for the next zoonotic disease outbreak. Accurate parametrization of these models requires data from diverse sources, including the scientific literature. Despite the abundance of scientific publications, the manual extraction of these data via systematic literature reviews remains a significant bottleneck, requiring extensive time and resources, and is susceptible to human error. This study examines the application of a large language model (LLM) as an assessor for screening prioritisation in climate-sensitive zoonotic disease research. By framing the selection criteria of articles as a question–answer task and utilising zero-shot chain-of-thought prompting, the proposed method achieves a saving of at least 70% work effort compared to manual screening at a recall level of 95% (NWSS@95%). This was validated across four datasets containing four distinct zoonotic diseases and a critical climate variable (rainfall). The approach additionally produces explainable AI rationales for each ranked article. The effectiveness of the approach across multiple diseases demonstrates the potential for broad application in systematic literature reviews. The substantial reduction in screening effort, along with the provision of explainable AI rationales, marks an important step toward automated parameter extraction from the scientific literature.

## 1 Introduction

Zoonotic diseases are becoming increasingly more prevalent due to increased interactions among, humans, livestock, wildlife, disease vectors, and pathogens, exacerbated by climate change and rapid human expansion into natural habitats [1,2]. Globally, zoonotic diseases have disproportionately impacted impoverished livestock workers in low- and middle-income countries, and are responsible for millions of human deaths every year [3]. To address this threat, there is a mounting need for systems to forecast and model the spread of diseases, thereby aiding public health planning and supporting early warning systems [1].

Constructing effective disease models depends on precise parametrisation, which requires data from multiple sources, including clinical records, environmental datasets, grey literature, and scientific publications [4,5]. A systematic literature review (SLR), considered the gold standard [6], is used to reliably extract and synthesise this information. The key stages of the SLR process consist of planning and developing protocols, searching, screening articles for relevance, extracting information, assessing the quality of studies, and, finally, synthesising and reporting [7,8].

Despite the reliability of SLRs, the manual process demands significant time and resource investment [9], it incurs substantial financial costs [10], and is difficult to scale given the exponential growth of publications [11]. This inefficiency creates a major bottleneck, resulting in outdated reviews, unnecessary waste, and potential harm in decision making [12].

Conducting SLRs in multidisciplinary fields, such as climate-sensitive zoonotic diseases, introduces further challenges. Research in this area spans diverse domains including epidemiology, ecology, veterinary sciences, and public health [13,14]. Identifying the relevant literature requires broad search terms and careful screening, as studies may focus on indirect indicators to assess disease risk, such as disease vector distribution or environmental factors like rainfall, temperature, humidity, and extreme weather events [15–17], rather than direct pathogen detection. Furthermore, confounding factors such as vector control, antimicrobial treatment, and infrastructure changes obscure the assessment of climate impact [18], rendering the selection of primary studies a cognitively intensive task.

Automation offers a cost-efficient, faster, and more reliable solution, capable of meeting the demands for scale and quality [19–21]. Among the most promising advancements in this domain are large language models (LLMs) based on the transformer architecture [22], which have gained significant popularity [23,24]. These models leverage vast amounts of text data and advanced contextual understanding to handle complex language tasks, including screening citations [25–29], extracting data [29–31], and synthesising information [29,32,33]. However, integrating LLMs into SLR workflows poses major challenges [24]. They can produce plausible-sounding erroneous responses known as hallucinations [34], promote negative biases seen in training data [35], lack transparency, and are considered *“black-box”* systems [36]. Furthermore, comprehensive evaluation of LLMs must consider diverse factors including language tasks, reasoning, robustness, trustworthiness, and ethics [37]. While these issues are significant, researchers are actively exploring various options to mitigate risks [36–39]. Targeted applications of LLMs in specific SLR tasks offer promising opportunities to enhance efficiency while minimising risks.

One effective way to leverage LLMs in the SLR workflow is through relevancy ranking for screening prioritisation, the process of ranking the most relevant studies to streamline the screening stage. Title–abstract screening is the most time-consuming aspect of the manual SLR process [40]. Best-practice guidelines recommend that primary studies are sorted by relevance before being screened [41]. This can improve reviewer efficiency, increase productivity, and facilitate faster decision making, especially for highly skewed datasets, such as those in the medical domain, which constitute only 3% to 6% [42] of relevant documents. The output of a relevancy ranking system can be directly utilised by selecting the top-k ranked articles based on an approximation of relevant document coverage [43,44] or, alternatively, assist users in selecting *seed* or example articles for input into a further specialised binary classifier tool.

Current methods for automated screening prioritisation can be categorised into query-based and model-based approaches [45]. Query-based approaches determine relevancy ranking via a query generated from attributes of the review protocol, including title, Boolean queries, or selection criteria. These methods are often grounded in traditional information retrieval techniques, such as BM25 [46]. Model-based approaches utilise classification models trained to distinguish between relevant and irrelevant documents, often employing techniques such as active learning and relevance feedback [45].

Both query-based and model-based approaches have proven effective in studies of encoder-only BERT-based large language models [43,45,47,48]. However, these models require computationally intensive fine-tuning within the operational domain [43,45,47] and present challenges in understanding the rationale behind their ranking decisions. In contrast, generative large language models, like the GPT series, which utilises only the decoder layer of the transformer architecture [49], exhibit advanced natural language understanding and can perform zero-shot task solving while providing explanations through their language generation capabilities, as demonstrated in recommendation systems [50]. Despite these advantages, their application has mostly been explored in SLR binary classification research [25,51–53] and remains under-explored for SLR screening prioritisation [54,55], particularly in domain-specific applications.

While query-based methods have demonstrated effectiveness in automated screening prioritisation, the success of these approaches heavily depends on the values used in the query, which are derived from the SLR eligibility criteria. These criteria constitute a series of rules and specific requirements that a document must satisfy for inclusion in the SLR. Typically, these criteria are evaluated based on research question or inclusion/exclusion criteria, but could also be framed as a question-answering problem [56]. The Question-Answering (QA) framework, a best-practice screening tool in manual SLR workflow [41], provides a fine-grained, consistent, and targeted approach to determining eligibility. The integration of a QA framework has demonstrated promising results in previous research when applied to screening prioritisation [55], while also offering a mechanism to capture model reasoning, thereby enhancing transparency and interpretability in the decision-making process. The capacity to capture model reasoning and improve transparency has frequently been overlooked in prior research.

To address these challenges, this research proposes leveraging a generative LLM for relevancy ranking for screening prioritisation in climate-sensitive zoonotic diseases through a QA framework. This approach aims to enhance the SLR workflow while capturing model responses and investigating its utility for improving transparency and interpretability for human reviewers.

### 1.1 Contribution

This paper contributes to enhancing information retrieval techniques using generative LLMs, advancing the automation of SLRs and investigating explainable AI methods for ranking and reviewing the literature on climate-sensitive zoonotic diseases. The specific contributions can be summarised as follows:

- Validating the use of a generative LLM for relevancy ranking of primary studies by utilising a QA framework in the area of climate-sensitive zoonotic diseases.
- Evaluating how well the solution can generalise across the climate-sensitive zoonotic disease literature.
- Evaluating the utility of the LLM-generated reasoning text for human reviewers to enhance transparency and trust.

#### 1.1.1 Problem Definition

The focus of this study is to investigate the utility of using an LLM to rank primary studies by relevance utilizing a QA-framework. Given a set of documents 𝒟 and a set of eligibility criteria 𝒞, the task of the relevancy ranker is to assign each document *d* ∈ 𝒟 a relevance score *r*_*d*_ ∈ [0, 1], where *r*_*d*_ indicates how well document *d* satisfies the criteria 𝒞. A score closer to 1 indicates higher relevance, while a score closer to 0 indicates lower relevance.

Using the QA framework approach, Kusa et al. describes the eligibility criteria 𝒞 being transformed into a set of questions *𝒬* = {*q*_1_, · · ·, *q*_|*C*|_}, where *q*_*k*_ corresponds to a specific criteria *C*. A set of predicated answers ${\hat{A}}^{d}=\left\{{\hat{a}}_{k}^{d}|meets\left(q_{k},{\hat{a}}_{k}^{d}\right)\right\}$ can be obtained for each document *d* ∈ 𝒟′, where $meets\left(q_{k},{\hat{a}}_{k}^{d}\right)$ denotes that the document *d* should meet the criterion expressed by *q*_*k*_. The relevancy score ${\hat{r}}_{d}$ of a document can be computed on the predicate answers ${\hat{A}}^{d}$ using an aggregation method such as linear weighted sum (LWS).

#### 1.1.2 Background

There are multiple ways to use LLMs for ranking documents; rankers [43,54,57,58] and assessors are [59] popular choices. Rankers determine the order of items based on their perceived value, while assessors provide an evaluation or judgement of the quality or suitability of a single item.

Zero-shot rankers [50], have no need for prior training or examples and can be categorised into point-wise [58], which scores one query and document at a time, ranking the documents based on a score; pair-wise, where the model assesses a pair of documents against a query; or list-wise [57], which involves presenting both query and the complete list of items in the prompt. Pair-wise and list-wise methods do not scale for long lists, as is the case with SLRs where the initial search could yield a substantial list of results. Wang et al. examined the performance of zero-shot and fine-tuned point-wise neural rankers in the context of SLR document ranking and discovered that zero-shot neural rankers performed worse than traditional methods like BM25 in the absence of fine-tuning. Moreover, research utilising point-wise rankers with generative LLMs indicated that obtaining ranking scores from log-likelihood values resulted in superior ranking performance relative to employing LLM-generated labels [58]. The potential for additional output from an LLM, such as reasoning text, is constrained in this approach, as each output token, the smallest text unit a model can process, must be individually parsed, ultimately increasing complexity and limiting the interpretability of the ranker. Such constraints and the reliance on fine-tuning for improved performance highlights the need for approaches that balance scalability and transparency in zero-shot settings.

Zero-shot LLM assessors [59] perform relevancy judgement on a query-document pair when provided with a set of relevancy labels. When ground truth labels are available, these assessors are frequently employed to generate annotated datasets [60]. In addition to issuing judgement, LLM assessors can offer natural language explanations to support their conclusions; they provide scalability, consistency, and the potential to complement human assessors in judgement tasks [59].

Several studies investigating the application of LLMs in SLR workflow have highlighted the importance for human oversight [25,32,51,53] or the use of LLMs as assistant reviewers [25,61]. Adopting an LLM as an assessor can effectively realise these strategies. While there are no ground truth data available at the start of a review, the assessor’s approach can still assign labels based on predefined eligibility criteria, establishing an initial framework to guide the ranking process and provide transparency. This study examines the effectiveness of an LLM as an assessor only, utilising its capacity to provide answer labels to questions.

Both ranker and assessor approaches can be enhanced using various strategies ranging from resource intensive options, such as pre-training and fine-tuning of models [43,62], to prompt engineering [38]. Domain-specific pre-training and fine-tuning have demonstrated substantial performance gains [33,43,63]. However, techniques such as few-shot and chain-of-thought (CoT) prompting, leveraging a *persona*, and adopting fine-grained labels offer a more economical, less complex initial approach prior to pursuing advanced optimisations. While zero-shot prompting does not utilise task-specific examples, few-shot prompting incorporates both positive and negative examples, demonstrating promising results in title–abstract screening automation [27,62,64,65]. Considering that ranking occurs at the very start of the screening process, users typically lack domain-specific examples at this early stage, making few-shot prompts less practical for an initial ranking task. Chain-of-thought (CoT) prompting instructs the model to adopt a grounded, *“step-by-step”* approach to task resolution, reducing the likelihood of *hallucinations*, improving performance and accuracy [38]. To increase transparency, this study captures the CoT reasoning so users can gain insights into the model reasoning. Additionally, integrating a *persona* can enhance a model’s capacity to deliver more tailored and consistent responses across multiple interactions, adapting to a wide range of scenarios [51,66]. Finally, recent experiments with zero-shot LLM rankers indicate that fine-grained relevancy labels help guide the model to differentiate documents more effectively [58]. This study specifically examines the impact of fine-grained labels on the ranking performance using an LLM as an assessor within a zero-shot setting for prioritising primary studies.

### 1.2 Research Questions

This study aims to evaluate the effectiveness of using a generative LLM in the role of an assessor to assist in the relevancy ranking of primary studies in climate-sensitive zoonotic diseases. To guide the investigation, the following research questions are posed:

**RQ1** How does an LLM-based assessor utilising a QA framework compared to baseline models utilising review title and selection criteria?

**RQ2** Does the label granularity effect the ranking performance of an LLM-based assessor utilising a QA framework for climate-sensitive zoonotic disease?

**RQ3** Does the ranking performance of an LLM-based assessor generalise across climatesensitive zoonotic disease datasets with varying relevance rate?

**RQ4** Does CoT rationale provided by an LLM assist a human reviewer’s ability to detect misclassifications in SLR?

## 2 Methodology

A series of experiments was conducted to investigate the research questions outlined in Section 1.2. This section provides details of the dataset, models, the evaluation metrics, and the experimental design.

### 2.1 Dataset

The dataset used in this study consists of 2905 title–abstracts related to four zoonotic diseases: Crimean–Congo haemorrhagic fever (CCHF), Ebola virus, Leptospirosis (Lepto), and Rift Valley fever virus (RVF), together with one climate variable, rainfall. Each combination of disease and climate variable constitutes a distinct SLR. An SLR protocol was established, outlining the selection criteria for each combination of disease and climate variable. A search was conducted in the PubMed and Scopus online journal repositories and the results were imported into a reference manager, along with titles and abstracts followed by de-duplication. A team of 5 researchers (early and mid-career researchers, postgraduate student, and undergraduate student) were trained to screen abstracts using the criteria outlined in Table 1. Abstracts that met the inclusion criteria were assigned a score of 1; otherwise a score of 0. When a reviewer was unsure, abstracts were discussed with one of the mid-career researchers who had experience in reviewing infectious diseases climate sensitivity. Abstracts that were unclear, or did not precisely meet the study selection criteria, were scored 1 to be further examined in the full-text inspection stage.

Table 2 illustrates the composition of the dataset. Ebola publications exhibited a particularly pronounced skew, with only 1.5% (14/915) deemed relevant, while the remaining diseases ranged from 10.8% to 12.6%. The titles–abstracts were exported to a CSV file along with the manually assigned scores, and labels were assigned per record to indicate the target disease and climate variable (rainfall).

### 2.2 QA Framework

Following the abstract screening best-practice guideline [41], a set of eligibility questions was developed to evaluate the relevance of title–abstracts based on the selection criteria. Distinct sets of eligibility questions were formulated for each disease and the associated climate variable (rainfall), ensuring their relevance to the SLR topic, as detailed in Table 3. It should be noted that reviewers did not use these questions during their screening, as they were developed retrospectively for the evaluation process. The guidelines stipulate that the questions should be clear and concise, and must be (1) objective, (2) “single-barrelled” or focused on a specific aspect of the citation, (3) use a consistent sentence structure, and (4) ensure responses are limited to yes, no, and unsure only. Furthermore, the questions should be organised hierarchically, starting with the easiest and progressing to difficult. In this study, the question text and the number of questions assigned to each disease remained constant across experiments, while the answer labels were varied. The specific variations are detailed in Section 2.4.

### 2.3 Prompts

The design and choice of prompting has implications on the model response [61,67]. The prompts deployed in this study were inspired by those from previous research [25,27,38,51,61,68] and received iterative refinement. The prompt performance was evaluate using *recall@k* and mean average precision *MAP* metrics detailed in Section 2.7.

A persona description stating *“You are a world leading expert veterinary epidemiologist screening abstracts of scientific papers for the systematic literature review of ‘$topic’”* was established prior to the main instruction prompt. All experimental runs used the same persona, with the *$topic* placeholder dynamically replaced by the topics listed in Table 3.

Two main instruction prompts were designed for this study. The first prompt, TSC prompt, serves as a baseline, utilising the review title and selection criteria. This prompt is an adaptation of the *Zero-shot Framework CoT* prompt [27], directing the model to analyse the title and apply inclusion and exclusion criteria to classify a given abstract as “Definitely Include”, “Probably Include”, “Probably Exclude”, “Definitely Exclude”, or “Unsure” (see Listing 1). The placeholders *$title, $inclusion, $exclusion*, and *$abstract* were populated dynamically according to the disease context of the experiment.

Listing 1 **TSC prompt, based on the review title and selection criteria**


1 Task: You are a researcher rigorously screening titles and abstracts of scientific papers for inclusion or exclusion
  ↪ in a review paper titled “$topic”. The following is an excerpt of two sets of criteria. A study is considered
  ↪ included if it meets all the inclusion criteria. If a study meets any of the exclusion criteria, it should be
  ↪ excluded. Here are the two sets of criteria:
2
3 Inclusion criteria:
4 $inclusion
5
6 Exclusion criteria:
7 $exclusion
8
9 Abstract:
10 “$abstract”
11
12 We now assess whether the paper should be included from the systematic review by evaluating it against each and
  ↪ every predefined inclusion and exclusion criterion. First, we will reflect on how we will decide whether a paper
  ↪ should be included or excluded. Then, we will think step by step for each criteria, giving reasons for why they
  ↪ are met or not met.
13
14 We will conclude by outputting: “Definitely Include”, “Probably Include”, “Probably Exclude”, “Definitely Exclude”
  ↪ or “Unsure”.
15
16 Required Format:
17 Format the output as a JSON object with the following keys.
18     “reason”: Step-by-step reasoning to the question.
19     “answer”: “Definitely Include”, “Probably Include”, “Probably Exclude”, “Definitely Exclude” or “Unsure”.
20 Example Format:
21 {
22     “reason”: “<YOUR REASONING FOR THE QUESTION>“,
23     “answer”: “<YOUR FINAL ANSWER>“
24 }
25
26 Strict Output Requirements:
27 You MUST NOT output any other text before or after the JSON.
28 Do NOT be chatty. Output exactly what is instructed.

The second prompt, QA prompt in Listing A1 (see Appendix A), utilises the QA framework discussed in Section 2.2. This prompt instructs the model to follow a structured approach to answering the eligibility questions with respect to a given abstract. The answer labels available to the model are discussed in Section 2.4. Similar to the first prompt, placeholders for *$abstract* and *$question* are dynamically populated with title–abstract and eligibility questions based on disease context.

Both prompts employ predefined answer labels and explicit reasoning, adhering to the principles of CoT prompting [38], and align with the *instructive* template category, as described in [65]. Given that GPT models are *autoregressive* [62], where previous tokens influence the final answer, the prompts were designed to explicitly request reasoning prior to delivering a final answer label. Finally, the prompts instruct the model to format the response as a JSON object; fields capture the reasoning text, answer, and, in the case of the QA framework, the question number. This structure helps ensure consistent and reliable output formatting and eliminates the need for parsing natural text while facilitating easy integration with downstream components.

### 2.4 Answer Labels

Table 4 lists the predefined answer schemas designed to be used with the QA and TSC prompt, restricting the model to selecting only from these options. The schema QA-3 was adapted from the best-practice guidelines [41], while QA-4 and Qa-5 were inspired by [58].

Additionally, a variant of the QA-2 schema, termed QA-2-C, a multi-level answer schema, was explored by instructing the model to generate a separate confidence score (Table 5). This method aims to more accurately capture the uncertainty that the original QA-2 fails to account for, yet appears in the other schemas. Each answer label was assigned a predefined answer score to quantify its contribution to the relevancy ranking calculation (described in Section 2.5); in the case of QA-2-C, the confidence score was used in place of the answer score. The QA prompt was adjusted to accommodate the multi-level scoring, as shown in Listing A2 (see Appendix A).

### 2.5 Relevancy Ranking

A relevancy score was computed for each title–abstract record by employing a linear weighted sum (LWS) of the *answer scores* derived from all questions within the QA framework. The records are subsequently sorted in descending order based on the relevancy score. In this study, it was assumed that all questions carried equal weight. Formally, let

- *𝒬* = {*q*_1_, · · ·, *q*_|*C*|_} be the eligibility questions derived from the selection criteria.
- ${\hat{A}}^{d}=\left\{{\hat{a}}_{k}^{d}\right\}$ be the set of predicated answers for each question *q*_*k*_ in document *d*.
- *w*_*k*_ be a predefined weight reflecting the importance of each question *q*_*k*_.

Then, the relevancy score ${\hat{r}}_{d}$ of document *d* is expressed as

(1) $${\hat{r}}_{d}=\sum_{k=1}^{\left|C\right|}w_{q}{\hat{a}}_{k}^{d}$$

where, for equal-weighted questions, $w_{k}=\frac{1}{k}$ for all *q*. This naturally simplifies to

(2) $${\hat{r}}_{d}=\frac{1}{k}\sum_{k=1}^{\left|C\right|}{\hat{a}}_{k}^{d}$$

In cases where ties occured in the relevancy score, title–abstract length was used as a tiebreaker by sorting the title–abstracts in descending order of length, assuming that longer articles were more relevant.

### 2.6 Models

#### 2.6.1 Baseline Models

The BM25 and MiniLM v2 [69] models were utilised to establish a zero-shot baseline. BM25 is a widely used ranking algorithm used in information retrieval; it evaluates a document’s relevance with respect to a query. The query in this instance is a concatenation of the review title with the selection criteria. BM25 scores were computed for each title–abstract and query pair following a preprocessing step that included lower casing, punctuation elimination, stop word removal, and stemming. The results were ranked according to the BM25 scores, with the text length serving as a tie-breaker.

MiniLM v2 is a BERT-based, pre-trained sentence-transformer model optimised for embedding text, and it is trained on a massive and diverse dataset [70]. This study used the *all-MiniLM-L6-v2* model without fine-tuning. Embedding vectors were generated for both title–abstracts and queries. Cosine similarity was computed as per Equation (3) for each vector pair, and subsequently normalised and sorted, with the text length serving as a tie-breaker.

(3) $$\;cosine\_similarity\;(d,q)=\frac{d\cdot q}{\left\|d\|\|q\right\|}$$

This methodology for establishing a baseline is aligned with the approach implemented by CSMed, a meta-dataset comprising 325 systematic literature reviews from the medical and computer scientific fields [56].

#### 2.6.2 Large Language Models

For this experiment, *GPT-4o-mini-2024-07-18*, a smaller and optimised version of *GPT-4*, was utilised to achieve a balance between cost, performance, and computational efficiency [71]. The model was used in its pre-trained form without any fine-tuning and accessed via the *OpenAI API*. The sampling *temperature* value was set to 0 to improve reproducibility and ensure deterministic responses between invocations but also to ensure that it did not affect the model’s ability to *self-correct* [67]. The *max_token* parameter was set to 512 to provide sufficient context for processing the prompts and to generate concise reasoning responses. The *response_format* parameter was used to ensure consistent and reliable output formatting. A JSON Schema was assigned to this parameter that describes the structure of the expected output, preventing the need for parsing of natural text and facilitating integration with downstream code.

### 2.7 Evaluation Metrics

The performance of ranking tasks in information retrieval challenges is evaluated using rank-based metrics and metrics at predefined cut-offs, such as the *top-k*% of retrieved documents [56,72]. The evaluation metrics used to measure the performance of the approach are listed below:

#### Recall @ k%

*Recall*, also known as *sensitivity*, provides a measure of how many relevant documents are retrieved within the *top-k*% of ranked results.

#### nWSS @ r%

Normalized work saved over sampling (nWSS), equivalent to *true negative rate* (or *Specificity*), quantifies the effort or work saved by the automated system when compared to random sampling, assuming a fixed *recall* level, and can be utilized to evaluate outcomes across models and datasets [73]. Here *nWSS* is evaluated at *r* = 95% and *r* = 100%.

#### AP

Average precision (*AP*) represents the average of precision computed at each relevant document’s position, considering all documents retrieved up to a specific rank. It combines both *precision* and *recall*, evaluating how effective documents are ranked by relevance. Unlike *precision @ k%*, which is influenced by the total number of relevant documents, *AP* addresses this limitation by providing a more balanced assessment [72].

#### MAP

Mean average precision (*MAP*) represents the average *AP* across individual information needs and provides a single robust metric for evaluating the ranking quality across *recall* levels [72].

### 2.8 Experimental Setup

The experiments were automated using Python 3.9.21, with each experimental run organised into separate Python scripts. The baseline experiments utilised title and selection criteria, abbreviated as TSC in conjunctions with BM25, MiniLM, and ChatGPT-4o-mini models. Meanwhile, the QA framework was exclusively tested with the ChatGPT-4o-mini model using all QA answer schemas list in Tables 4 and 5.

**Figure 1:**
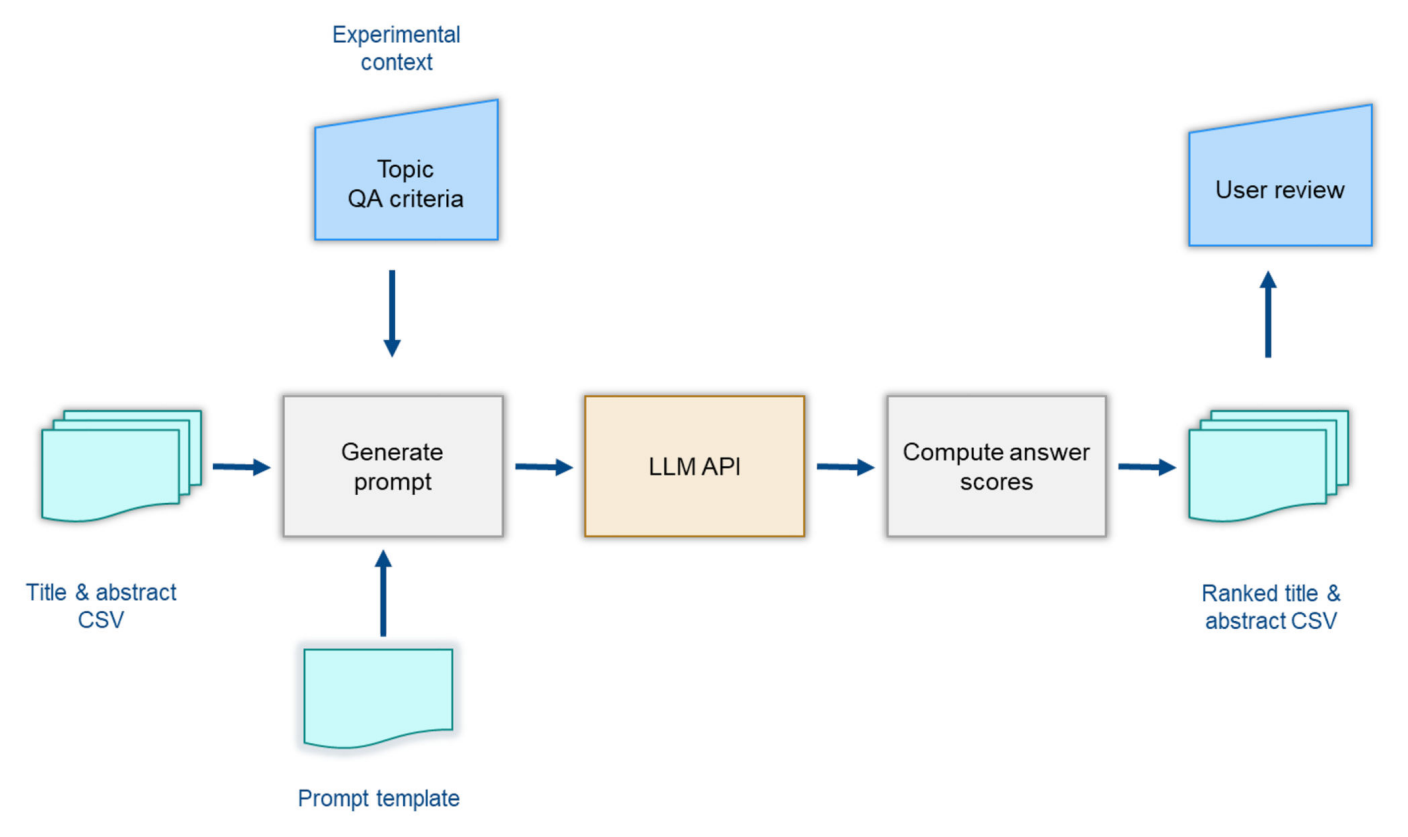
Overview of the experimental setup for title–abstract ranking using LLM-based QA framework.

Each execution script requires two input files: a CSV of title–abstracts and a prompt template file, which is dynamically populated with experimental context-specific values for each zoonotic disease: CCHF, Ebola, Lepto, and RVF (Figure 1). The code then calls the OpenAI API completion endpoint using predefined model parameters and the adapted prompts. By utilizing the API, the study ensures reproducibility. All responses from the API are captured and subsequently processed to calculate a relevancy ranking.

## 3 Results

This section presents the results of the eight models evaluated across the four diseases: CCHF, Ebola, Lepto, and RVF, evaluated against the study metric (Table 6).

The following sections will examine the results by each research question.

### 3.1 RQ1. LLM-Based QA Assessor vs. Baseline

For *RQ1*, the comparison is between QA-based models and the TSC baseline generative (TSC-5) and non-generative (TSC-BM25, TSC-MiniLM) models.

#### Recall@k%

**Figure 2:**
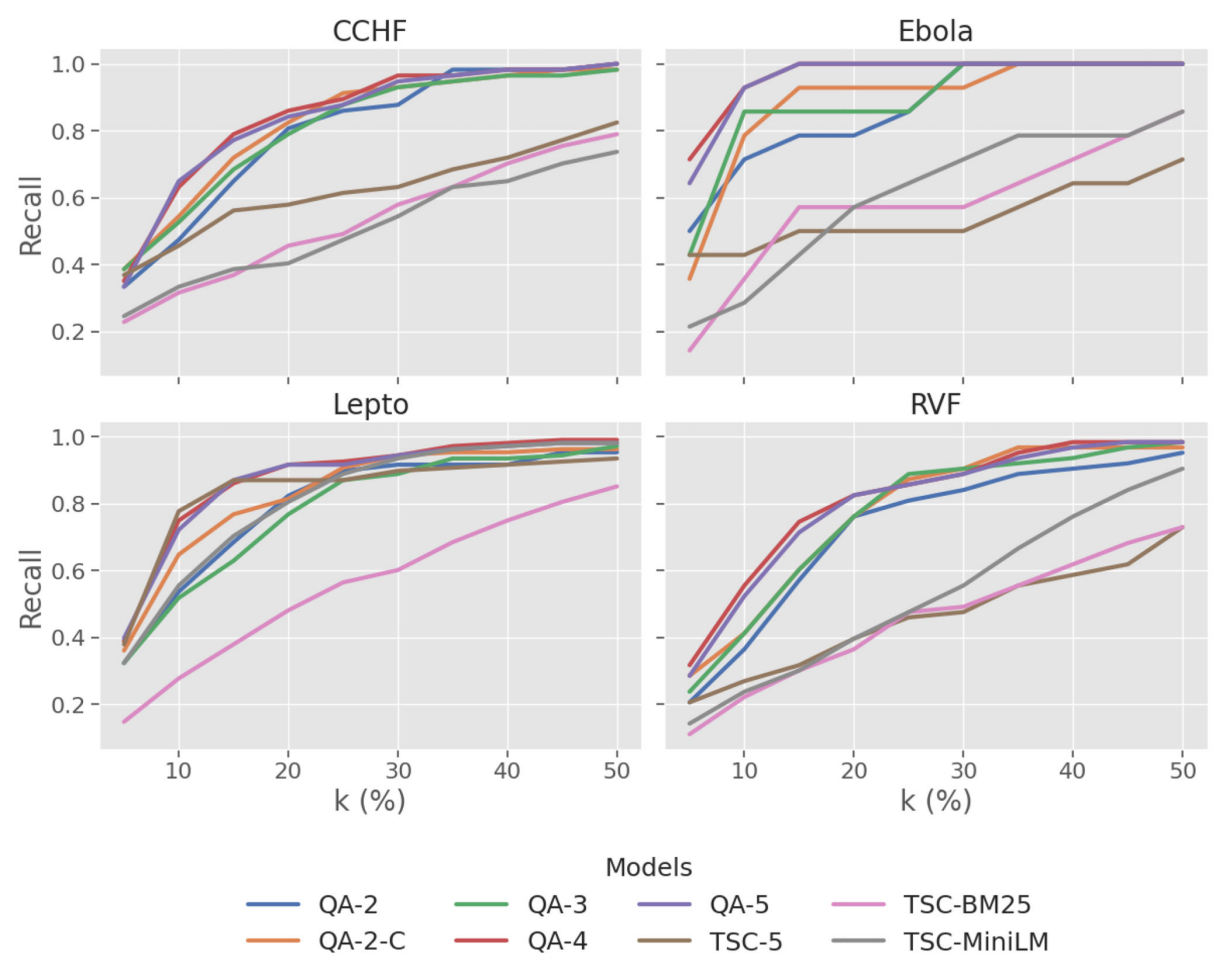
Plot of *recall* at varying levels of *k*% across four zoonotic diseases.

The results illustrated in Figure 2 exhibit that QA-based models consistently performed better than the TSC baseline models. At k = 50%, all QA-based models reached recall levels above 0.95. They also attained higher recall scores than the baseline in the early ranks k < 20%, particularly for Ebola.

#### nWSS@r%

**Figure 3:**
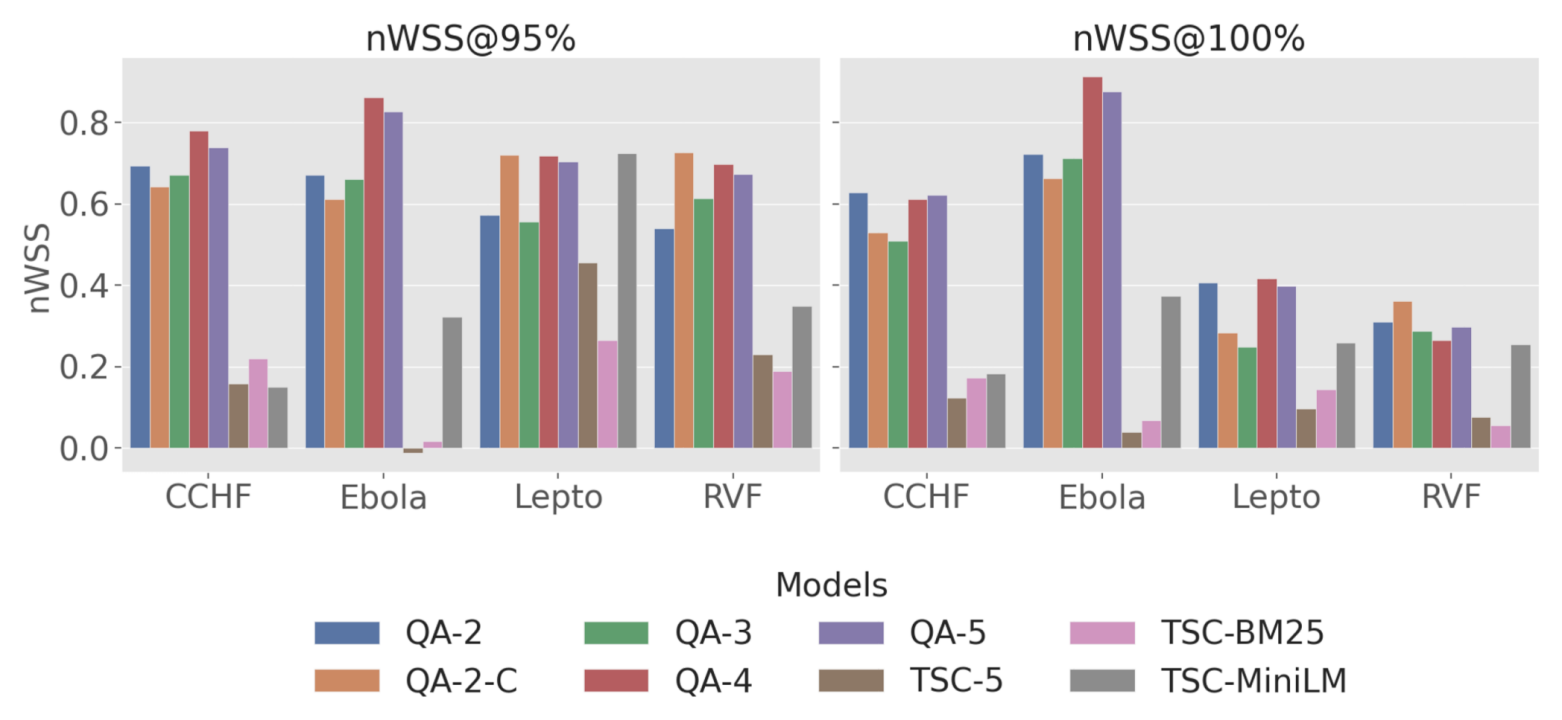
Plot of *nWSS* across four zoonotic diseases datasets at *recall* threshold of 95% and 100%.

In Figure 3, QA-4 showed the highest efficiency, especially for CCHF (0.78 at 95%), Ebola (0.86 at 95%), Lepto (0.72 at 95%), and RVF (0.70 at 95%), surpassing baseline models across all diseases. While the *nWSS* of QA-4 dropped when targeting r = 100%, it still remained superior to the baseline models. In contrast, the TSC models displayed lower overall work savings, with particularly high variability in the LLM-based TSC model: it ranged from negative savings with Ebola to 46% *nWSS@95%* with Lepto.

#### AP

**Figure 4:**
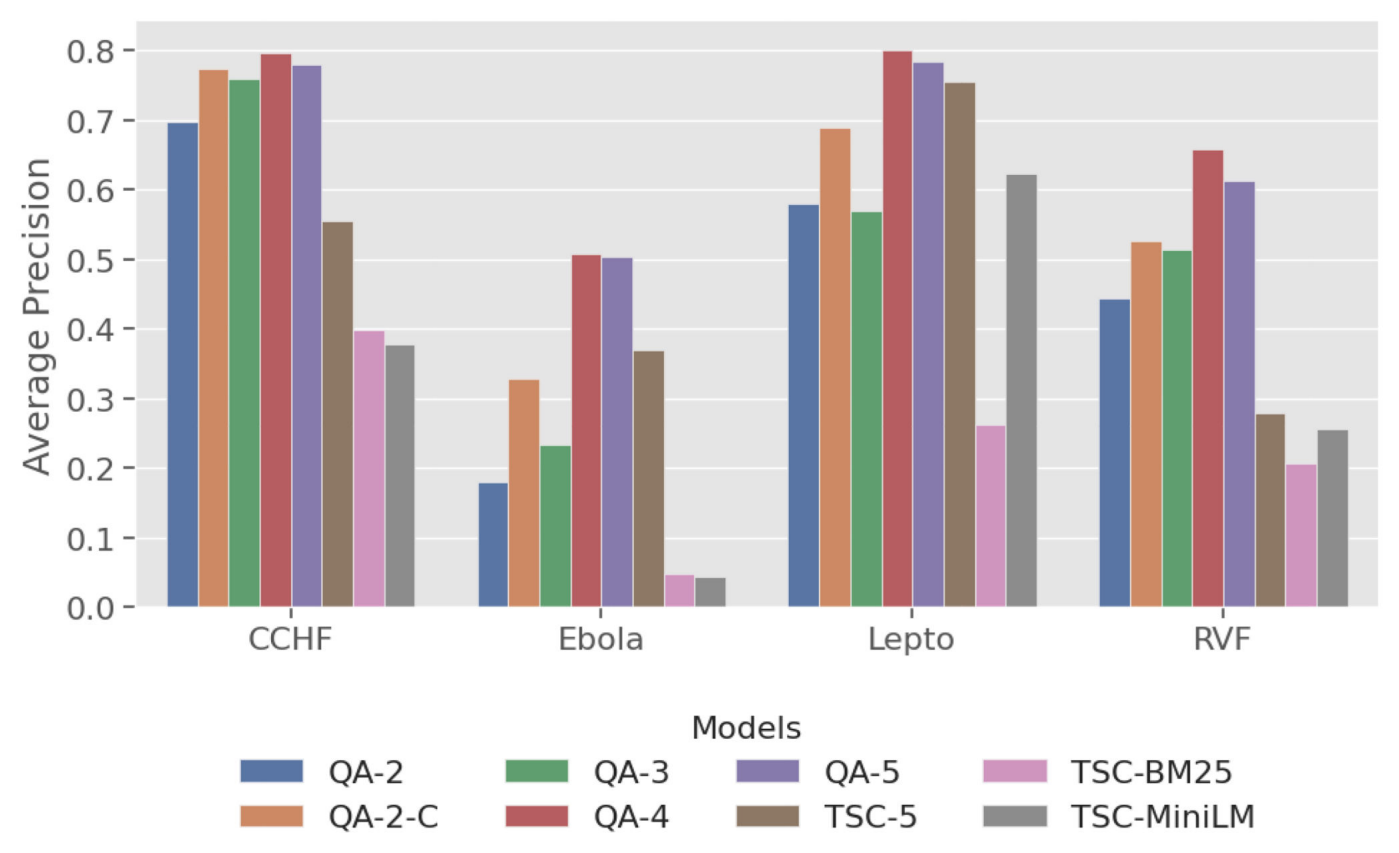
Plot of *average precision* across four zoonotic diseases.

This metric (Figure 4) evaluates ranking quality by measuring the number of relevant documents and how early they appear. The QA models, particularly QA-4, showed superior performance over the baseline models, with QA-4 achieving the highest AP values for all diseases except Ebola, where the performance was comparable to QA-5.

#### Ranking

**Figure 5:**
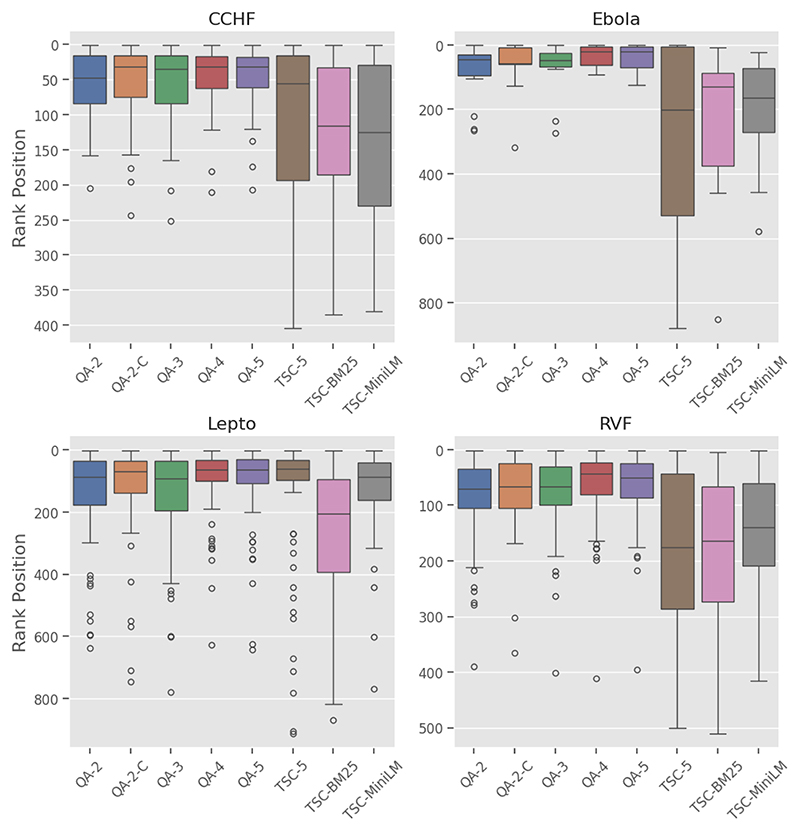
Plot of the distribution of rank positions of relevant title–abstract across four zoonotic diseases datasets.

The box plot in Figure 5 shows the distribution of the ranking positions for each model across the diseases. The QA models have narrower inter-quartile ranges (IQRs) and shorter whiskers, while the baseline models display higher medians and wider variability in rank positions.

#### MAP

**Figure 6:**
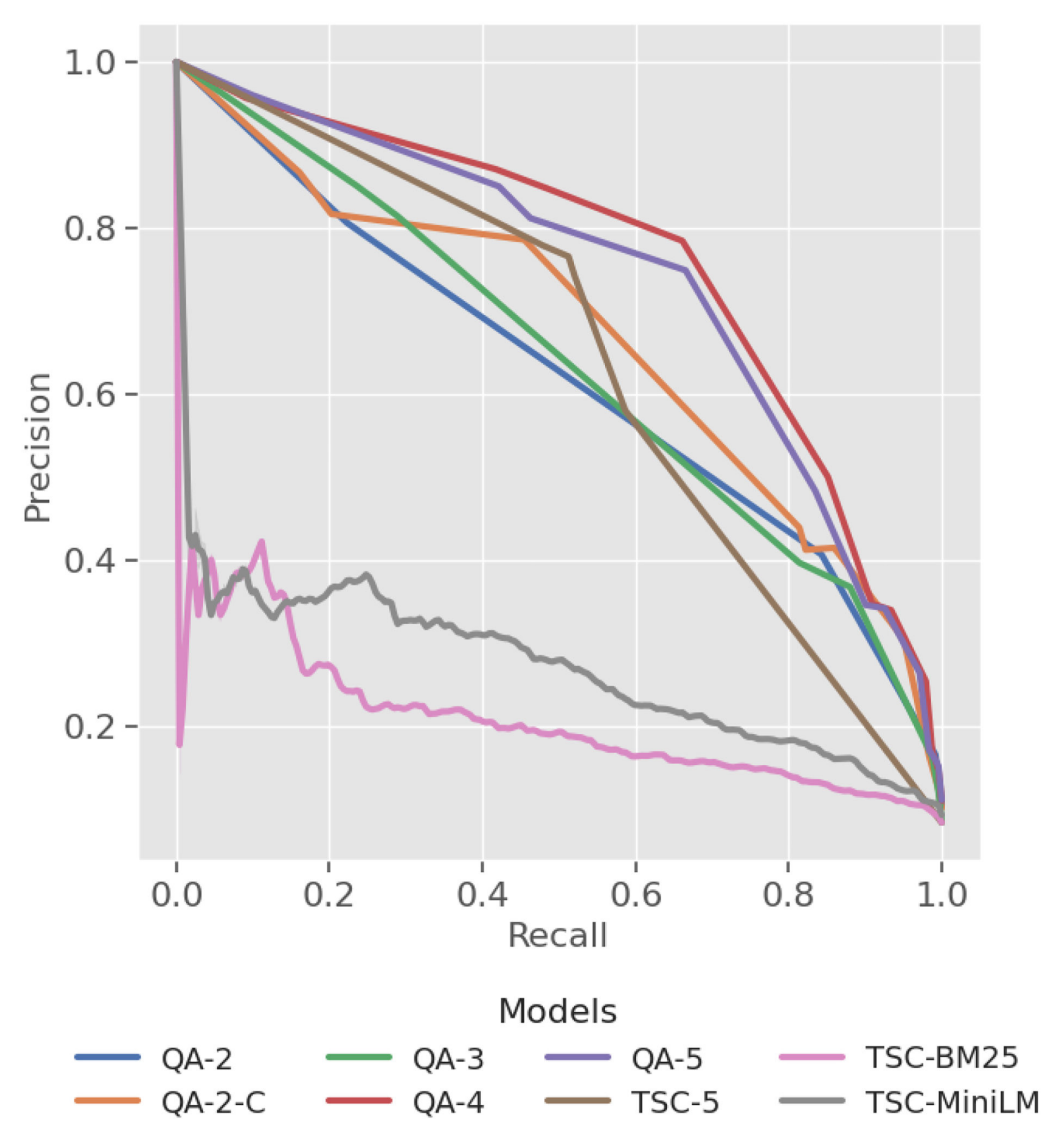
Plot of *precision* and *recall* across four zoonotic disease datasets.

The *MAP* metric in Table 7 offers a single performance measure for each model. The QA-4 model attained the highest *MAP* score of 0.691, while the baseline TSC-BM25 model had the lowest of 0.229. Notably, the TSC-5 model achieved a 0.489, beating the QA-2 model. A *precision–recall curve* (Figure 6) visualises overall performance by illustrating the effect of varying thresholds on the precision–recall balance. The QA models exhibit a larger area under the PR curve compared to the baseline models, although the TSC-5 model is positioned closer to the QA model curve in the precision–recall space than those of TSC-BM25 and TSC-MiniLM.

### 3.2 RQ2. Effect of Label Granularity

This section analyses performance across the QA-based models. QA-2 and QA-3 are categorised as single-level coarse-grained label models, while QA-4 and QA-5 are categorised as single-level fine-grained label models. QA-2-C is identified as a multi-level label model.

#### Recall@k%

In Figure 2, generally, *recall* improves with granularity (QA-2 to QA-4), with diminishing returns observed from QA-4 to QA-5. Single-level fine-grained labels (QA-4, QA-5) exhibit high *recall* at early ranks (k < 20%). By k = 50%, there is not much difference as they reach near complete *recall* (≥ 0.98) across all disease datasets.

#### nWSS@r%

Results for *nWSS@r%* indicate that QA-2-c, QA-4, and QA-5 exhibit significant work savings at 95% *recall* target over coarse-grained labels (QA-2, QA-3), achieving a minimum of 61%, 70%, and 67%, respectively. In particular, QA-4 and QA-5 exhibit exceptional savings on highly skewed datasets such as Ebola, realising 83% to 88% savings at *recall* levels of 95% and 100%.

#### AP

Fine-grained labels tend to lead to higher precision, with QA-4 consistently achieving the highest AP scores across diseases, particularly for Lepto and RVF, with QA-5 not far behind.

#### Ranking

Fine-grained label models (QA-4, QA-5) are positioned higher in the plot, indicating lower ranking, and consistently have lower medians across all diseases when compared to the other models.

#### MAP

The *MAP* scores in Table 7 demonstrate a clear trend, indicating improved ranking performance with increased label granularity but peaking at QA-4 (0.691). This is also evident in the *precision–recall curve*, where QA-4 maintains higher precision across most recall values compared to QA-5, which trails slightly below it in the precision–recall space.

### 3.3 RQ3. Performance Across Zoonotic Diseases

This subsection examines performance variations across all models (QA and TSC) and diseases (Ebola, Lepto, CCHF, RVF).

#### Recall@k%

When looking across zoonotic disease datasets, QA-based models maintain a *recall* level between 0.95 and 1.00 at k = 50%. In the Ebola dataset, QA-4 and QA-5 models demonstrate a significantly high *recall*, achieving complete *recall* at *k* = 15%. The baseline models did not achieve a comparable level of recall, with BM25 exhibiting a consistent but significantly lower recall across all diseases. In contrast, TSC-5 and TSC-MiniLM performed well with Lepto but severely unperformed with other diseases.

#### nWSS@r%

For all diseases, the QA models retain a minimum of 54% work saving at *nWSS@95%*; however, performance drops when transitioning to *nWSS@100%* for all disease except Ebola, where savings slightly increase. Notably, TSC-MiniLM achieves the same work savings as QA-4 and QA-2-C (*nWSS@95% = 0.72*); however, when striving for full *recall*, this value reduces dramatically, proving high variability.

#### AP

A consistently high *AP* across diseases indicates models that can handle diseasespecific terminology and generalise well independently of relevancy rates. The leading QA models (QA-4, QA-5) achieved a minimum of 0.5 *AP* across all diseases while the baseline models exhibit lower and greater variability in AP.

#### Ranking

QA models displayed the best rank distribution with the Ebola dataset (Figure 5), demonstrated by the lower median rank, tighter IQR, and few outliers. Similar patterns were seen in Lepto, CCHF, and RVF; however, it is important to note that the Lepto dataset has the highest number of outliers.

### 3.4 RQ4. Utility of Generated CoT Rationale

The CoT reasoning logic proved to be consistent, in line with human expectations, helpful in detecting edge cases, and capable of revealing variations in misclassifications across disease datasets. Listing 2 is an example RVF response from the QA-4 model that demonstrates the logic that the model provides for each title–abstract record. A senior reviewer on the team was presented with the results to manually assess the validity of the responses. For each disease, title–abstracts with relevancy scores exceeding a predetermined threshold and classified as irrelevant, as well as those below the threshold classified as relevant, were displayed in MS Excel alongside the answer scores and reasoning text for each question. The reviewer did report any significant issues in the model’s reasoning and considered the explanatory text highly beneficial for comprehending the logic underlying the answer scores. Using the sort and filter facilities provided in MS Excel, the reviewer was able to efficiently review and identify several misclassified items, as shown in Table 8. Lepto (16), RVF (13), and CCHF(10) had the most misclassifications, highlighting the inherent variability in manual SLR screening quality but also opportunities to refine the selection criteria and eligibility questions. These findings not only highlight the potential of CoT reasoning to ensure transparency in the SLR process but also highlight a means to iteratively enhance the ranking process.

Listing 2 **Example JSON response for an RVF abstract**


 {
1     “results”: [
2         {
3                  “question_number”: 1,
4                    “reason”: “The abstract describes a study that identifies mosquito larvae species and examines their
5                    ↪ habitats, indicating that it is based on original data collection rather than a review or
6                    ↪ meta-analysis.”,
			“answer”: “Definitely Yes”
7          },
8          {
9                    “question_number”: 2,
10                    “reason”: “The study discusses mosquito species that are vectors for diseases like Rift Valley Fever but
                    ↪ does not measure the incidence or prevalence of the virus itself, focusing instead on the mosquito
                    ↪ larvae.”,
11                    “answer”: “Probably No”
12        },
13        {
14                    “question_number”: 3,
15                    “reason”: “The research includes surveys conducted during dry and rainy seasons, which implies an
                    ↪ examination of seasonal environmental factors that could impact vector distribution.”,
16                    “answer”: “Definitely Yes”
17        },
18        {
19                    “question_number”: 4,
20                    “reason”: “The study involves field surveys and ecological assessments rather than laboratory method
                    ↪ validation, focusing on real-world data collection.”,
21                    “answer”: “Definitely Yes”
22        }
23      ]
24   }

## 4 Discussion

This study explored the use of a generative LLM as an assessor for screening prioritisation in climate-sensitive zoonotic disease research. Findings revealed that a zero-shot LLM-based QA assessor, leveraging fine-grained labels, can effectively rank primary studies by relevance and demonstrates broad generalisability across four climate-sensitive zoonotic disease datasets with differing relevancy rates. Additionally, the CoT reasoning text generated by this approach provided valuable insight to human reviewers and aided in the identification of misclassified records in the disease datasets.

Before further interpretation of the results, the following important limitations are noteworthy. Most importantly, the study depended on a single reviewer for the manual evaluation of the model’s reasoning text. No metrics were established to measure the quality or potential bias of the text generated by the LLM, which are prone to *“hallucination”* [34,35]. Nevertheless, the results from the study demonstrated a potential use case for such data and the possibility for creating a unique dataset to enable the team to conduct further research into reasoning and bias within LLM-based approaches.

This study used a single closed-source commercial LLM from OpenAI to conduct all experiments. Restricting the evaluation solely to ChatGPT-4-o-mini restricts the generalisation to other LLMs. Additionally, while a fixed model version was used with the *temperature* parameter set to 0 to ensure deterministic behaviour, and prior research confirming consistent outcomes on repeated invocation [61], ongoing optimisations by OpenAI may have led to performance changes that could have influenced the results. In the future, the team intends to explore additional models, including open-source alternatives applying the same methodology.

Moreover, the eligibility questions used in the experiments were generated in retrospect following manual screening; hence, there is a risk of overfitting and misalignment with broader applicability. Polanin et al. advocates for conducting a pilot screening session with a subset of abstracts to refine and validate eligibility questions prior to screening. This approach could ensure clarity and consistency of the questions while improving the effectiveness of the rankings. Such an exercise may prove beneficial for future screening efforts, as it could mitigate bias and facilitate the development of a more comprehensive dataset. Additionally, the collection of ground truth data at the question level would facilitate a more precise and detailed evaluation of the model and support further research.

Lastly, although the real-world setting of this study’s dataset demonstrated the effectiveness of the approach, it has not been thoroughly evaluated on datasets from other domains. CSMed is an initiative to create a standardised dataset to evaluate the performance of automated SLR screening models [56], a gap highlighted in recent research [24]. It provides access to several hundred SLRs in the medical and computer science domain and presents an opportunity to benchmark the approach proposed in this study and gain a broader understanding of its performance, generalisability, and adaptability across domains.

Despite the limitations, this study demonstrates the effectiveness of employing a QA framework with an LLM-based assessor to robustly rank the literature on four climate-sensitive zoonotic diseases by relevance. The QA models consistently outperformed all the baseline models, achieving high *recall@k%* and *MAP* scores across all disease datasets in this study. High *recall* is crucial in SLR automation systems [74] to prevent bias and ensure that all relevant articles are identified, while a high *MAP* score reflects strong discrimination and resilience to imbalanced datasets [72], ensuring that reviewers are presented with both manageable and highly relevant articles. Additionally, the high *nWSS@r%* values (Figure 3) obtained by the top QA models demonstrate their ability to detect irrelevant documents early in the screening process, thereby reducing the number of items requiring manual review. The leading QA models (QA-4) demonstrate a minimum savings of 70% at *r = 95%* across all diseases; however, performance declines when aiming for complete recall. Lastly, the QA models exhibit less variability and more consistency across the datasets compared to the baseline models, as evidenced in Figure 5 by the narrower inter-quartile ranges.

While the study results are promising, it is important to contextualise the system performance. Direct comparisons to other systems must be made with caution due to the challengingly unique climate-sensitive zoonotic disease dataset employed in this study. However, the baseline TSC-BM25 model provides a simple yet informative reference point. In the study by Wang et al. examining the performance of neural rankers on the CLEF [75] datasets, a baseline BM25 model recorded a *recall@20%* ranging from 0.52 to 0.64, alongside an *MAP* score of 0.16, using title as the input query to the model. By comparison, TSC-BM25 in this study recorded a *recall@20%* ranging from 0.37 to 0.57 and a slightly higher *MAP* score of 0.23 using title and selection criteria as the input query. Their most effective fine-tuned BioBERT neural ranker demonstrated a *recall@20%* ranging from 0.82 to 0.89, and an *MAP* score of 0.381. In contrast, the QA-4 model in this study attained a *recall@20%* ranging from 0.83 to 1.0, and a significantly higher *MAP* score of 0.670. Although the highly skewed Ebola dataset may have contributed to the elevated recall score, the findings highlight the potential of the zero-shot LLM-based QA framework for enhancing ranking performance without the need for fine-tuning. However, any conclusions should be deferred until a comprehensive evaluation with a standardised benchmarking dataset is conducted.

The adoption of a QA framework has been shown to be highly effective in the current study, contrary to the findings of prior research such as Kohandel Gargari et al., where a screening tool approach yielded poor performance. Upon examining their methodology, several factors may have contributed to the discrepancy, including the complexity of the prompt, ambiguity in managing “unclear” labels, and the assumption that the model will adhere to the embedded logic flow in the prompt without errors. Additionally, their research employed GPT 3.5, an older model that is less advanced and less accurate. In contrast, the findings from Akinseloyin et al. align more closely with this study, utilising a similar QA framework methodology. However, their eligibility questions were derived from the inclusion/exclusion criteria using an LLM and observed that the generated questions lacked complete independence and recommended that such questions be created by humans for better reliability, a recommendation followed in this study.

In addition to the QA framework, the use of fine-grained labels appears to have a consistent positive impact on ranking tasks, whether in an assessor or a ranker context. The single-level fine-grained models, QA-4 and QA-5, demonstrated superior recall, precision, and work savings, with minimal variability, compared to the coarser-grained models, QA-2 and QA-3. Fine-grained labels appear to enhance the system performance by supplying the model with broader answer options, allowing it to more effectively represent uncertainty. However, the benefits diminished as granularity increased beyond four levels, as evidenced by the reduced *MAP* score for QA-5. Zhuang et al. found that fine-grained relevancy labels improved performance, with no advantage in exceeding four levels of granularity using a point-wise LLM-based ranker. While the tasks in these studies differ (assessor vs. ranker), this suggests that fine-grained labels may be broadly beneficial for LLM-based ranking tasks.

Interestingly, the shift from a single-level to a multi-level labelling approach (QA-2-c), which pairs binary labels with confidence scores and demonstrates a middle ground between fine-grained (QA-4, QA-5) and coarse-grained (QA-2, QA-3) labels, resulted in a reduced performance. Several factors could account for this outcome. Firstly, the multi-level model employs a more intricate prompt; simple prompt structures are more effective than complex ones [76]. Furthermore, a single-level classification inherently captures both the relevance and uncertainty in one consolidated label (e.g., “Probably Yes”), whereas a two-step classification introduces uncertainty post-decision, thereby splitting the context. Finally, the model’s autoregressive characteristic [62], combined with single versus multiple decision-making points, may account for the observed performance differences.

The study approach also shows promising generalisability across climate-sensitive zoonotic disease datasets, despite their varied characteristics. The Ebola dataset reveals a significant skew, with only 1.5% of relevant records, whereas the CCHF, Lepto, and RVF datasets demonstrate moderate skews, as outlined in Table 2. This variation is apparent when evaluating model performance across the different diseases. Yet, QA-based models, specifically QA-4 and QA-5, retained higher *recall@k%, nWSS@95%*, and *AP* scores while maintaining consistent ranking across the datasets in comparison to the baseline models. The QA models also maintained the best rank distribution across all diseases (Figure 5), demonstrated by the lower median rank and tighter IQR, suggesting that QA models are superior to baseline models at prioritising relevant articles in the earlier ranks. Nevertheless, the Lepto dataset has the greatest number of outliers, which may be a sign of ambiguous abstracts, misaligned classification criteria, or model limitations.

Nicholson Thomas et al. used a selection-criteria-based prompt, similar to the TSC-5 baseline model, to screen articles for ecosystem condition indicators. They report that to handle multidimensional topics with high precision, iterative refinement of the selection criteria was essential. While further enhancements of the selection criteria could enhance the performance of the TSC-5 model for diseases where it underperformed (other than Lepto), the QA framework offers an additional layer of flexibility. It decouples the decision making from the LLM’s internal logic by decomposing the assessment into granular questions. Weak signals in the form of uncertainty labels (e.g., “Probably No” or “Unsure”) allow studies that marginally fail to still contribute to overall ranking through the LWS. This flexibility ensures that relevant studies are not prematurely excluded, and provides adaptability to the varied disease datasets.

Complementing the flexibility of the QA framework is the utility of reasoning text generated through CoT prompting, which provides a detailed explanation for each relevancy assessment, exposing the models’ decision-making processes to the reviewers. For example, the reasoning text in Listing 2 illustrates the model’s ability to justifying uncertain responses like “Probably No” in question 2, where it explains that although the study addresses disease vectors, it does not directly assess virus prevalence. On the spectrum of human–machine collaboration, this approach of automatically generating a first-pass judgement with rationale falls into the *human verification* category, or *human-in-the-loop* approach [59]. While CoT prompting does not ensure the accuracy of the reasoning path, it does enable the model to more effectively access relevant data learned during pre-training [38], which may explain the QA models’ performances over the baseline models. Further investigation is required to assess the validity and reliability of the generated rationale.

Finally, the approach proposed in this study can currently be used as a standalone decision aid to reduce the risk of human error and bias during the initial screening phase. The main prompt provided in the supplementary code is generic and applicable across domains. All that is needed is to formulate research-specific eligibility questions, define the review topic, and modify the persona text accordingly. Data are output as a CSV file containing ranking scores, answers, and reasoning text, and can be reviewed in a tool such as MS Excel, providing an additional layer of adaptability. By prioritising and streamlining the review process, this approach allows human reviewers to focus on the edge cases, making it a practical and efficient solution for SLRs.

## 5 Conclusions

This study reports the empirical results of a relevancy ranking approach that leverages an LLM as an assessor, guided by a QA framework, to rank the literature on four climate-sensitive zoonotic diseases and one climate variable (rainfall). The findings demonstrate that an LLM-based QA assessor using zero-shot CoT prompting can effectively and reliably rank primary studies by relevance across four zoonotic disease datasets with varying relevance rates. Notably, fine-grained QA models significantly outperform the baseline models, achieving strong *recall* at multiple thresholds, high *MAP* scores, and substantial work savings of at least 70% (NWSS@95%). Additionally, the CoT reasoning text generated by this approach provides valuable insight, assisting researchers to identify several misclassified items and enhancing transparency and confidence in the screening process.

Although further empirical research is necessary to validate the approach against standardised benchmark datasets, the substantial reduction in screening effort, combined with the provision of explainable AI rationales, represents an important step toward automated parameter extraction from the scientific literature.

## Supplementary Material

Appendix

## Figures and Tables

**Table 1:** Selection criteria used in the systematic review for the abstract screening process.

Inclusion Criteria	Exclusion Criteria
Primary research or meta-analysis Assesses the relationship between the selected climate variable and either:	
– Disease incidence or prevalence. – Pathogen survival. – Transmission. – Virulence. – Demonstrated vector or maintenance host survival, development or distribution.	Reviews, opinions, books, editorials. Laboratory-focused studies, e.g., studies to develop an appropriate culture method.

**Table 2:** Overview of dataset composition by disease, climate variable, and relevance proportions.

Disease	Climate Variable	Relevant	Irrelevant	Total	% Relevant
CCHF	Rainfall	57	397	454	12.6%
Ebola	Rainfall	14	901	915	1.5%
Lepto	Rainfall	108	891	999	10.8%
RVF	Rainfall	63	474	537	11.7%

**Table 3:** Disease-specific topics and question-based eligibility criteria for climate-sensitive zoonotic disease studies.

Disease	Topic and Eligibility Questions
CCHF	**Topic**: Impact of Climate Change on CCHF: A Focus on Rainfall **Eligibility Questions**: Q1. Does the study report on primary research or a meta-analysis rather than a review, opinion, or book? Q2. Does the study measure the incidence or prevalence or virulence or survival or transmission of Crimean-Congo haemorrhagic fever or a relevant vector (such as ticks) without specifically measuring the incidence of the pathogens? Q3. Does the research examine environmental factors such as rainfall, seasonality (e.g., wet vs. dry season) or regional comparisons impacting disease prevalence or vector distribution? Q4. Is the study focused on field-based or epidemiological research rather than laboratory method validation?
Ebola	**Topic**: Impact of Climate Change on Ebola: A Focus on Rainfall **Eligibility Questions**: Q1. Does the study report on primary research or a meta-analysis rather than a review, opinion, or book? Q2. Does the study measure the incidence or prevalence or virulence or survival or transmission of Ebola or Marburg, a relevant vector, or reservoir hosts abundance or distribution (such as bats or primates) without specifically measuring the incidence of the pathogens? Q3. Does the research examine environmental factors such as rainfall, seasonality (e.g., wet vs. dry season) or regional comparisons impacting disease prevalence or vector distribution? Q4. Is the study focused on field-based or epidemiological research rather than laboratory method validation?
Lepto	**Topic**: Impact of Climate Change on Leptospirosis: A Focus on Rainfall **Eligibility Questions**: Q1. Does the study report on primary research or a meta-analysis rather than a review, opinion, or book? Q2. Does the study measure the incidence or prevalence or virulence or survival or transmission of Leptospirosis, a relevant arthropod vector, or reservoir hosts (such as rodents) without specifically measuring the incidence of the pathogens? Q3. Does the research examine environmental factors such as rainfall, seasonality (e.g., wet vs. dry season) or regional comparisons impacting disease prevalence or vector distribution? Q4. Is the study focused on field-based or epidemiological research rather than laboratory method validation?
RVF	**Topic**: Impact of Climate Change on Rift Valley Fever Virus: A Focus on Rainfall **Eligibility Questions**: Q1. Does the study report on primary research or a meta-analysis rather than a review, opinion, or book? Q2. Does the study measure the incidence or prevalence or virulence or survival or transmission of Rift Valley fever or other vector-borne diseases (such as malaria) that share similar vectors (e.g., mosquitoes) without specifically measuring the incidence of the pathogen? Q3. Does the research examine environmental factors such as rainfall, seasonality (e.g., wet vs. dry season) or regional comparisons impacting disease prevalence or vector distribution? Q4. Is the study focused on field-based or epidemiological research rather than labora-tory method validation?

**Table 4:** Answer labels and scoring scales for single-level QA and TSC models.

Answer Schema	Answer Labels	Answer Score
QA-2	Yes	1.0
	No	0.0
QA-3	Yes	1.00
	Unsure	0.50
	No	0.00
QA-4	Definitely Yes	0.95
	Probably Yes	0.75
	Probably No	0.25
	Definitely No	0.05
QA-5	Definitely Yes	1.00
	Probably Yes	0.75
	Unsure	0.50
	Probably No	0.25
	Definitely No	0.00
TSC-5	Definitely Include	1.00
	Probably Include	0.75
	Unsure	0.50
	Probably Exclude	0.25
	Definitely Exclude	0.00

**Table 5:** Answer labels and scoring scales for multi-level QA models.

Answer Schema	Answer Labels	Confidence Labels	Confidence Score
QA-2-C	Yes	High	1.00
		Medium	0.75
		Low	0.50
	No	High	0.00
		Medium	0.25
		Low	0.50

**Table 6:** Performance metrics for models by zoonotic diseases.

Disease	Model	Recall@k%					nWSS@r%		AP
		5	10	20	30	50	95	100	
CCHF	QA-2	0.33	0.47	0.81	0.88	1.00	0.69	0.63	0.70
	QA-2-C	0.39	0.54	0.82	0.93	0.98	0.64	0.53	0.77
	QA-3	0.39	0.53	0.79	0.93	0.98	0.67	0.51	0.76
	QA-4	0.35	0.63	0.86	0.96	1.00	0.78	0.61	0.80
	QA-5	0.33	0.65	0.84	0.95	1.00	0.74	0.62	0.78
	TSC-5	0.37	0.46	0.58	0.63	0.82	0.16	0.12	0.55
	TSC-BM25	0.23	0.32	0.46	0.58	0.79	0.22	0.17	0.40
	TSC-MiniLM	0.25	0.33	0.40	0.54	0.74	0.15	0.18	0.38
Ebola	QA-2	0.50	0.71	0.79	1.00	1.00	0.67	0.72	0.18
	QA-2-C	0.36	0.79	0.93	0.93	1.00	0.61	0.66	0.33
	QA-3	0.43	0.86	0.86	1.00	1.00	0.66	0.71	0.23
	QA-4	0.71	0.93	1.00	1.00	1.00	0.86	0.91	0.51
	QA-5	0.64	0.93	1.00	1.00	1.00	0.83	0.88	0.50
	TSC-5	0.43	0.43	0.50	0.50	0.71	-0.01	0.04	0.37
	TSC-BM25	0.14	0.36	0.57	0.57	0.86	0.02	0.07	0.05
	TSC-MiniLM	0.21	0.29	0.57	0.71	0.86	0.32	0.37	0.04
Lepto	QA-2	0.32	0.54	0.82	0.92	0.95	0.57	0.41	0.58
	QA-2-C	0.36	0.65	0.81	0.94	0.96	0.72	0.28	0.69
	QA-3	0.32	0.52	0.77	0.89	0.97	0.56	0.25	0.57
	QA-4	0.39	0.75	0.92	0.94	0.99	0.72	0.42	0.80
	QA-5	0.40	0.72	0.92	0.94	0.98	0.70	0.40	0.78
	TSC-5	0.38	0.78	0.87	0.90	0.94	0.46	0.10	0.75
	TSC-BM25	0.15	0.28	0.48	0.60	0.85	0.26	0.14	0.26
	TSC-MiniLM	0.32	0.56	0.81	0.94	0.98	0.72	0.26	0.62
RVF	QA-2	0.21	0.37	0.76	0.84	0.95	0.54	0.31	0.44
	QA-2-C	0.29	0.41	0.76	0.90	0.97	0.73	0.36	0.53
	QA-3	0.24	0.41	0.76	0.90	0.98	0.61	0.29	0.51
	QA-4	0.32	0.56	0.83	0.89	0.98	0.70	0.27	0.66
	QA-5	0.29	0.52	0.83	0.89	0.98	0.67	0.30	0.61
	TSC-5	0.21	0.27	0.40	0.48	0.73	0.23	0.08	0.28
	TSC-BM25	0.11	0.22	0.37	0.49	0.73	0.19	0.06	0.21
	TSC-MiniLM	0.14	0.24	0.40	0.56	0.90	0.35	0.26	0.26

**Table 7:** Results for mean average precision (MAP) and area under the precision–recall curve (PRAUC) by model.

Model	MAP	PR-AUC
QA-2	0.476	0.621
QA-2-C	0.579	0.669
QA-3	0.519	0.636
QA-4	0.691	0.761
QA-5	0.670	0.740
TSC-5	0.489	0.639
TSC-BM25	0.229	0.206
TSC-MiniLM	0.325	0.271

**Table 8:** Misclassifications identified by the system.

Disease	Revised to Include	Revised to Exclude	Total
CCHF	7	3	10
Ebola	1	0	1
Lepto	13	3	16
RVF	8	5	13

## Data Availability

All of the code used in this study is accessible in a github repository (link provided upon publishing).

## Abbreviations

The following abbreviations are used in this manuscript:

MDPI: Multidisciplinary Digital Publishing Institute
LLM: Large language model
QA: Question and answer
CCHF: Crimean–Congo haemorrhagic fever
RVF: Rift valley fever
