# Supplementary material for: Accelerating Disease Model Parameter Extraction: An LLM-Based Ranking Approach to Select Initial Studies For Literature Review Automation
Source: Mach Learn Knowl Extr. Author manuscript; Available in PMC 2026 Apr 4. (PMC7618976; doi:10.3390/make7020028)
Supplement: Appendix [file EMS212926-supplement-Appendix.pdf]

Appendix A. Additional Prompts and Disease Information

Listing A1. QA single-level prompt, based on QA framework.

```
Task: Analyse the abstract below within the double quotes and answer the questions below. Take a step-by-step approach towards reasoning and then answer the questions with either [ANS-SCHEMA-LABELS] only.
Keep the reasoning short and concise and do not repeat the question.

Abstract:
"$abstract"

Question:
$questions

Required Format:
Format the output as a JSON object with a single key, "results", containing an array of objects. Each object should represent an answer to a specific question.

Answer Requirements:
Answer all questions without exceptions.
Do not add any text before or after the JSON output.

Object Structure:
Each object within "results" should contain the following fields:
    "question_number": Number of the question.
    "reason": Step-by-step reasoning to the question.
    "answer": [ANS-SCHEMA-LABELS]

Example Format:
{
    "results": [
        {
            "question_number": <THE NUMBER IN FRONT OF THE QUESTION YOU ARE ANSWERING>,
            "reason": "<YOUR REASONING FOR THE QUESTION>",
            "answer": "<YOUR FINAL ANSWER>"
        },
        # the next question number, answer and explanation
    ]
}

Strict Output Requirements:
You MUST answer all questions.
You MUST NOT output any other text before or after the JSON.
Do NOT be chatty. Output exactly what is instructed.
```

Listing A2. QA multi-level prompt, based on QA framework and supporting an answer and a confidence level.

```
Task: Analyse the abstract below within the double quotes and answer the questions below. Take a step-by-step approach towards reasoning and then answer the questions with either "Yes" or "No" only.
Keep the reasoning short and concise and do not repeat the question. Provide a confidence score to your answer reflecting how certain you are based on the provided context and your reasoning using the confidence scale below.

Confidence scale:
Low
Medium
High

Abstract:
"$abstract"

Question:
$questions

Required Format:
Format the output as a JSON object with a single key, "results", containing an array of objects. Each object should represent an answer to a specific question.

Answer Requirements:
Answer all questions without exceptions.
Do not add any text before or after the JSON output.

Object Structure:
Each object within "results" should contain the following fields:
    "question_number": Number of the question.
    "reason": Step-by-step reasoning to the question.
    "answer": "Yes" or "No"
    "confidence_score": Confidence score for your answer

Example Format:
{
    "results": [
        {
            "question_number": <THE NUMBER IN FRONT OF THE QUESTION YOU ARE ANSWERING>,
            "reason": "<YOUR REASONING FOR THE QUESTION>",
            "answer": "<YOUR FINAL ANSWER>",
            "confidence_score": "<YOUR CONFIDENCE SCORE FOR THE ANSWER>"
        },
        # the next question number, answer and explanation
    ]
}

Strict Output Requirements:
You MUST answer all questions.
You MUST NOT output any other text before or after the JSON.
Do NOT be chatty. Output exactly what is instructed.
```
